# Tissue Multiplex Analyte Detection in Anatomic Pathology – Pathways to Clinical Implementation

**DOI:** 10.3389/fmolb.2021.672531

**Published:** 2021-07-27

**Authors:** Keith A. Wharton, Douglas Wood, Mael Manesse, Kirsteen H. Maclean, Florian Leiss, Aleksandra Zuraw

**Affiliations:** Ultivue, Inc., Cambridge, MA, United States

**Keywords:** multiplex, digital pathology, whole slide image, tumor microenvironment, immunohistochemistry, immunofluorescence, pixel pathway, laboratory developed test

## Abstract

**Background:** Multiplex tissue analysis has revolutionized our understanding of the tumor microenvironment (TME) with implications for biomarker development and diagnostic testing. Multiplex labeling is used for specific clinical situations, but there remain barriers to expanded use in anatomic pathology practice.

**Methods:** We review immunohistochemistry (IHC) and related assays used to localize molecules in tissues, with reference to United States regulatory and practice landscapes. We review multiplex methods and strategies used in clinical diagnosis and in research, particularly in immuno-oncology. Within the framework of assay design and testing phases, we examine the suitability of multiplex immunofluorescence (mIF) for clinical diagnostic workflows, considering its advantages and challenges to implementation.

**Results:** Multiplex labeling is poised to radically transform pathologic diagnosis because it can answer questions about tissue-level biology and single-cell phenotypes that cannot be addressed with traditional IHC biomarker panels. Widespread implementation will require improved detection chemistry, illustrated by InSituPlex technology (Ultivue, Inc., Cambridge, MA) that allows coregistration of hematoxylin and eosin (H&E) and mIF images, greater standardization and interoperability of workflow and data pipelines to facilitate consistent interpretation by pathologists, and integration of multichannel images into digital pathology whole slide imaging (WSI) systems, including interpretation aided by artificial intelligence (AI). Adoption will also be facilitated by evidence that justifies incorporation into clinical practice, an ability to navigate regulatory pathways, and adequate health care budgets and reimbursement. We expand the brightfield WSI system “pixel pathway” concept to multiplex workflows, suggesting that adoption might be accelerated by data standardization centered on cell phenotypes defined by coexpression of multiple molecules.

**Conclusion:** Multiplex labeling has the potential to complement next generation sequencing in cancer diagnosis by allowing pathologists to visualize and understand every cell in a tissue biopsy slide. Until mIF reagents, digital pathology systems including fluorescence scanners, and data pipelines are standardized, we propose that diagnostic labs will play a crucial role in driving adoption of multiplex tissue diagnostics by using retrospective data from tissue collections as a foundation for laboratory-developed test (LDT) implementation and use in prospective trials as companion diagnostics (CDx).

## Introduction

In diverse human cultures, knowledge is disseminated by an esteemed individual who has achieved wisdom through discipline and sacrifice. Visualize the archetype: a wise sage, sitting on a mountain, legs folded, in meditative gaze. Advice seekers climb the mountain to pose their question or dilemma. Magic happens, wisdom is dispensed, and the seeker descends the mountain. From its origins in the mid-19th century to today, diagnostic anatomic pathology follows a similar construct. The foundation of knowledge is histopathology – examination of changes in cells and tissues viewed by microscopy to diagnose disease. The sage is the pathologist, who, through years of observation and discernment dispenses wisdom. Tissue samples and data workflows converge on the mountaintop, where the pathologist’s gaze is directed in a microscope. The “magic” is the pathologist’s integration, interpretation and judgment of data to establish a diagnosis that is reported in the medical record, codified in journals and textbooks, or simply Tweeted.

Rudolf Virchow, known as the father of histopathology, wrote that the body is like a state “*…in which every cell is a citizen. Disease is merely the conflict of citizens of the state…*” (Virchow, 1858). Diagnosis of disease, in particular cancer, is based on examination of cells by microscopy and on detection of specific molecules in cells. Proteins and nucleic acids are routinely identified in biopsy tissues by antibody-binding or nucleic hybridization technologies such as IHC and *in situ* hybridization (ISH). Nucleic acid amplification and sequencing technologies such as polymerase chain reaction (PCR) are routinely used in clinical practice to identify molecular alterations such as point mutations, chromosome translocations, and gene amplification/transcript overexpression. In the past decade, next generation sequencing (NGS) of hundreds to thousands of genes in parallel has entered clinical practice, increasing the efficiency of detection of abnormal genes that drive disease and impact treatment choices. Bulk transcript profiling of tissue samples over the past 2 decades has provided critical molecular insights into various cancers including lymphoma (Scott, 2015) and breast cancer (Perou et al., 2010), that have served as the basis of prognostic and predictive transcript signature tests such as OncotypeDX (Exact Sciences) (Siow et al., 2018). More recently, by deeply profiling each “citizen” involved in the conflict, single-cell profiling (transcriptomics, proteomics, etc.) has advanced understanding of cell phenotypes that drive disease, with implications for clinical practice (Marx, 2019; Aldridge and Teichmann, 2020). These data-rich sequencing and profiling techniques are powerful discovery tools, but for diagnostic use, the vast majority of data generated is extraneous and lacks the spatial context of histopathology. Nature’s 2020 Method of the year, spatially resolved transcriptomics, captures spatial context, but most of the methods do not have the cellular resolution of histopathology, the size and complexity of data remains largely beyond diagnostic comprehension, and the majority of the data produced will ultimately lack clinical utility (Marx, 2021). We hypothesize that multiplex immunofluorescence (mIF) will emerge as a leading technique that allows each pathologist, within their lab and scope of practice, to answer critical questions about disease diagnosis, prognosis, and prediction of response to the next generation of targeted therapies and their combinations, particularly in immuno-oncology (Tan et al., 2020).

### IHC and the Clinical Diagnostic Landscape

Despite increases in molecular diagnostic testing in recent years, IHC remains critical for histopathology diagnosis by revealing various molecular species *in situ* in a tissue sample. In IHC, antibodies against epitope(s) of a specific target (also referred to as a “marker” - most often proteins but also carbohydrates or nucleic acids – because they are used to mark cells) are applied to thin, formalin-fixed and paraffin embedded (FFPE) tissue sections mounted on glass slides. Slide pretreatment (“antigen retrieval”) breaks formalin cross links, allowing the antibody to diffuse into the tissue and bind mostly linear peptide (as opposed to conformational) epitopes (Sompuram et al., 2006). Bound antibodies are then detected with visualization reagents, most commonly secondary antibodies conjugated to the enzyme horseradish peroxidase (HRP). With peroxide, HRP converts soluble 3,3′-diaminobenzidine (DAB) into an insoluble brown precipitate that reflects antigen abundance and distribution in otherwise colorless tissue. Tissue structure is then visualized with a counterstain, typically hematoxylin, which labels, predominantly nuclei, a bluish-purple color. Robotic autostainers and optimized, prediluted reagents have improved speed and reproducibility of IHC in disease diagnosis (Grogan, 1992; Prichard, 2014). ISH to detect DNA is used to identify chromosomal translocations and gene copy number changes, and RNAscope (Advanced Cell Diagnostics, BioTechne Inc.) has emerged as a sensitive ISH technique to visualize RNA in FFPE tissue, offering advantages over traditional RNA ISH techniques to detect low abundance transcripts, and over antibodies in detecting some targets such as soluble cytokines or infectious agents (Wang et al., 2012; Carossino et al., 2020; Stein et al., 2021). The DAB/hematoxylin-stained slide is interpreted by a pathologist using a microscope, or increasingly, by viewing a scanner-generated whole slide image (WSI) on a computer monitor (Gurcan et al., 2009; Dimitriou et al., 2019).

How is IHC used in clinical diagnosis? Early IHC applications in the 1970s and 80s distinguished between major categories of neoplasia through demonstration, for example, that carcinomas express cytokeratins and lymphomas express leukocytic antigens, but not *vice versa* (Taylor, 1980; Debus et al., 1984). Over several decades the diagnostic roles for IHC have expanded to include: aiding in distinction between benign and malignant processes [e.g., p53 upregulation associated with malignancy (Yemelyanova et al., 2011), or p16 expression with HPV-positive squamous carcinomas (Shelton et al., 2017)], identification of specific cell types (e.g., CD68-positive macrophages, CD31-positive endothelial cells, Foxp3-positive regulatory T lymphocytes), subclassifying and refining diagnoses [e.g., association of marker positivity or negativity with histopathology-based differential diagnosis or molecular lesion, such as DNA mismatch repair deficient carcinomas (Wong et al., 2018) or BAP1-subtype melanomas (Shah et al., 2013)], providing information about disease drivers [e.g., C-myc translocated Burkitt’s lymphoma (Nwanze et al., 2017), N-myc amplified neuroblastoma (Santiago et al., 2019), EGFR-amplified cancers (Atkins et al., 2004)], allowing inference of various cell states and behaviors (e.g., Ki67 positivity and cell proliferation, Granzyme B positivity and activated cytotoxic T lymphocytes), activity of various growth stimulating and inhibiting pathways (Ras/MAPK, Hippo, Wnt/β-catenin, Hedgehog, Notch, TGF-β, and others), and assessment of predictive biomarkers associated with response to targeted therapies (Her2, ER/PR, PD-L1). While IHC may be capable of revealing a molecule of importance within tissue, whether it is the favored diagnostic modality is dependent on clinical context and test performance relative to other options such as FISH or NGS, such as is the case with detection of NTRK-family gene translocations that occur with low frequency in a wide variety of malignancies (Solomon et al., 2020).

Diagnostic IHC tests evolve. Pathologists refine IHC tests by testing new antibody clones, platforms, and detection reagents, and create new IHC tests based on markers discovered through research, diagnostic surveys, or to recapitulate other tests such as DNA mutation sequencing [e.g., mutation-specific antibodies such as BRAF V600E (Tetzlaff et al., 2015)] or transcript profiling [IHC panels to recapitulate subtypes of diffuse large B cell lymphomas (Yan et al., 2020a)]. Candidate IHC markers start in research laboratories—and most markers stay in research applications. A new marker can enter diagnostic practice through retrospective studies that demonstrate the marker’s improved utility over existing markers in defining a diagnosis or prognosis in a particular lesion type. Alternatively, the marker can enter diagnostic practice as the basis of a new standard of care through prospective investigations as a companion diagnostic (CDx) assay, as was the case with Her2 and PD-L1 IHC tests (Roach et al., 2016).

Because IHC tests are interpreted visually by a pathologist, marker choice and assay optimization is part science, part art – the intersection of truth and beauty. “Beauty” is ultimately subjective, assessed by signal strength, staining pattern, and signal to noise, whereas “truth” is assessed by biological plausibility, comparison of staining to reference standards (if they exist), and eliminating artifacts (Tsutsumi, 2021). Each IHC slide is typically scored for positivity or negativity of the tested marker in specific cell types; for cancer, whether the marker is present in cancer cells or the tumor microenvironment (TME) or both is assessed, always with reference to the location and appearance of different cell populations in the corresponding section stained with H&E. IHC CDx’s are usually scored in a semiquantitative fashion, based on marker distribution, percent of positive cells and/or stain intensity. However, it is important to note that IHC marker panels only aid in establishing a diagnosis of cancer; rather, it is the appearance of individual cells and overall tissue by H&E that forms the basis of a cancer diagnosis, with interpretation of a specific set of IHC stains helping to confirm, refine, or subclassify a diagnosis. For example, a lung cancer biopsy showing “carcinoma” on the H&E section is typically stained for a set of markers to determine whether it is best classified as adenocarcinoma or squamous carcinoma, as most adenocarcinomas will be positive for TTF1 and NapsinA, but negative for p63, and *vice versa* (Inamura, 2018). For carcinomas with unambiguous squamous, ductular, or other type of differentiation, IHC stains usually confirm the histological impression, but for the not uncommon tumor that displays few or paradoxical features of differentiation (e.g., epithelioid sarcomas, which display epithelial differentiation but express mesenchymal markers, or sarcomatoid carcinomas, which display mesenchymal differentiation but express epithelial markers), IHC marker panels are crucial for accurate, state of the art histopathology diagnosis (Huey et al., 2019; Czarnecka et al., 2020).

Today, most IHCs are used as an adjunctive to diagnosis, with specific markers chosen in groups or panels based on algorithms that aim to subclassify the lesion and answer diagnostic questions relevant to the specific clinical scenario (patient age, anatomic location), specimen type (skin, soft tissue), and histologic features of the H&E-stained tissue. In the United States, IHC of adjunctive markers poses a relatively low risk to patients because they are often used redundantly, as part of a panel or suggested diagnostic algorithm. Such algorithms are typically not standardized, with variation in algorithms across institutions and geographies attributed to variation in medical practice. Thus, a testing error - a false positive or false negative result - of a single IHC assay is unlikely to impact the final diagnosis. Accordingly, adjunctive IHC tests are classified by the United States Food and Drug Administration (FDA) in the lowest risk class (Class I) of *in vitro* diagnostics (IVD) (Medical Devices, 1998). A small but critical and growing set of markers, such as Her2, ER, PR, and PD-L1, predict (or, at best enrich for) response to specific therapies and are classified as companion diagnostics (CDx) (Scheerens et al., 2017). A related category of test, a complementary diagnostic, is similar to a CDx by providing useful predictive information, but is not required to administer a particular therapy (Scheerens et al., 2017). Predictive IHC markers are often single “high stakes” tests, errors in which entail a greater risk to patient safety, and are thus classified by FDA in the highest risk class (class III) of IVD (Jørgensen, 2016). While some benign and a few malignant diagnoses do not require any IHC, the current standard of diagnosis in 2021 for most malignant diagnoses, in particular a patient’s initial diagnosis, requires some IHC tests.

IHC used for patient diagnosis as a basis of medical decision making is regulated in the United States at a variety of levels. Assays must be validated and performed in a Clinical Laboratory Improvement Amendments (CLIA)-certified diagnostic laboratory, and be interpreted by qualified personnel such as a pathologist licensed to practice medicine in the state where the sample originates (Fetsch and Abati, 2010). Further lab certification by the College of American Pathologists (CAP) covers CLIA standards as well as assay performance assessment, proficiency testing, and adherence to specific practice guidelines such as processing and interpretation of breast cancer specimens (College of American Pathologists LEP, 2017). States such as New York have more rigorous laboratory standards and certification, enforced by the New York State Department of Health (NYSDOH) (New York State CLEP, 2021). A largely comparable but distinct international standard for diagnostic medical laboratories is ISO 15189 (Schneider et al., 2017). Laboratory tests are generally of two types: IVDs and Laboratory Developed Tests (LDT). While both test types require in-laboratory assay validation, IVDs are components and/or systems manufactured and distributed to laboratories for a specific purpose defined by an intended use statement, whereas LDTs are custom “single site” tests that may not be performed in laboratories other than where the test was developed. FDA regulates both IVDs and LDTs, but exercises enforcement discretion over most LDTs as constituting a part of medical practice, which is not regulated by FDA (Genzen, 2019). LDT and related regulation in the United States has been subject to attention and neglect over decades, and is not yet settled (Genzen et al., 2017). Recently, the United States VALID (Verifying Accurate, Leading-edge IVCT Development) Act, which proposes to classify all assays performed in diagnostic laboratories as *In Vitro* Clinical Tests (IVCT) and would allow the FDA to exert greater oversight of testing based on patient risk, has undergone several cycles of stakeholder feedback and revision (Konnick, 2020). In Europe, new legislation (IVDR) that applies to IVDs takes effect in May 2022, with implications for LDT development and practice (Bank et al., 2020; Stenzinger and Weichert, 2020). The language, interpretation, and enforcement of these regulations will impact global test development and deployment, particularly new tests based on innovative technologies, for decades to come (Huang et al., 2021).

Not all LDTs are equivalent, generally falling into two categories. A *de novo* or traditional LDT is a novel test “system” made from individual components, often sourced separately, each piece of equipment, input reagent, or other part of the assay system which may be labeled as an IVDs or for Research Use Only (RUO). A derived LDT is when a laboratory alters system components, instrument settings, or reaction conditions of an approved or cleared IVD, such that system definition and/or laboratory use deviates from the original product design and/or intended use statement of the parent IVD. If an LDT uses an IVD-labeled component, either type of LDT is considered an “off label” use of the IVD component. With IVDs, responsibility for test performance and thus risk of test failure is shared between the device manufacturer and the laboratory: the manufacturer is responsible for design, manufacturing, and performance of the IVD under defined conditions, and the laboratory is responsible for using the assay/device according to those conditions—only for its defined and specific purpose. In contrast, LDTs are the primary responsibility of the laboratory offering the test. Many IVD assays were first introduced to clinical practice as LDTs, so one key advantage of LDTs is the ability to quickly bring novel diagnostic technology to clinical practice. However, accompanying the lower barrier of LDTs to market entry is the possibility that poorly designed, developed, or performing tests may be used in patient care. Typically, the strict design, manufacturing, and testing requirements of IVDs are associated with more robust real-world product performance (such as accuracy, precision, multisite/multi-operator consistency, known failure modes with risk mitigation strategies in place) as well as market exclusivity that justifies a premium price or level of reimbursement. However, established IVDs can act as a barrier to rapid technological innovation by blocking competing technologies or companies who may offer superior technology or aspects of performance (e.g., lower cost, faster turn-around time, greater analytical sensitivity) but lack adequate clinical evidence to gain regulatory approval that drives adoption.

### Visualizing Multiple Markers

Cells of the immune system and the majority of hematolymphoid neoplasms are defined by coexpression of multiple cell surface markers, commonly assessed by flow cytometry. However, the vast majority of IHCs used in clinical diagnosis of solid tumors interrogate a single marker per tissue section (termed “singleplex”) and are thus unsuited to characterize cell phenotypes defined by coexpression of multiple markers when those markers are in the same subcellular compartment. In current diagnostic practice, many cases require multiple IHC markers, and the pathologist examines one marker at a time, one slide at a time, noting which cell populations on the slide are positive vs. negative for each marker then integrating the results to establish a final diagnosis. Occasional cases, particularly undifferentiated solid tumors and lymphomas, require more than two dozen different markers to arrive at a proper diagnosis, making the task of tallying and interpreting IHC results challenging. For such tumors, molecular profiling is playing an increasingly important diagnostic role (Yan et al., 2020b). For tumors in which sampling may be restricted to fine needle aspirates (FNA) or core needle biopsies (CNB) that yield limiting tissue, using multiple consecutive sections for singleplex IHC as well as possibly splitting the biopsy for diagnostic molecular testing such as PCR or NGS can compromise accurate diagnosis. For example, the majority of lung cancer patients present with advanced disease that is not amenable to surgical intervention, so diagnostic, prognostic, and predictive factors must be obtained from FNA or CNB of a mediastinal lymph node, or even a “liquid biopsy” (NGS to detect circulating tumor DNA in a peripheral blood sample) (Chen and Zhao, 2019). For such cases, multiplex staining allows visualization of all markers of interest using a minimal number of tissue sections.

By performing sequential or simultaneous chromogenic IHC staining reactions on a single slide, it is possible to generate multiplex chromogenic stained slides (Morrison et al., 2020). However, there are only limited diagnostic scenarios where chromogenic multiplex staining is currently used. These are situations in which the pathologist has greater confidence in a diagnosis when two markers labeling different cell populations or tissue compartments are present in the *same slide* than when the same two markers are present in *different slides* of the same tissue block. Given the availability of >1 enzyme to detect antibodies in chromogenic IHC [in addition to HRP, alkaline phosphatase (AP) is commonly used], and various enzyme substrates with distinct absorption spectra, two or more staining reactions can be performed sequentially, creating two or more different colors (plus a counterstain), each color representing a different marker or cocktail (mixture) of markers. Such assays typically require more complex test development and validation to ensure the multiplex staining reactions recapitulate the performance of each singleplex marker. For labs requiring fast assay turn-around time, duplex assays take longer to develop, cost per slide is typically higher than an equivalent number of singleplex reactions, and, at least in the United States, reimbursement mechanisms to incentivize use do not exist. One widely used stain is “PIN4,” which labels, in different colors, benign and malignant cell populations on the same slide to help the pathologist distinguish *in situ* from invasive cancer (Tacha and Miller, 2004). Most chromogenic multiplex assays are developed to label separate cell populations (e.g., cancer vs. non-cancer cells) or cell structures (plasma membrane vs. nucleus of same cell), without the intention of assessing marker colocalization, and, like adjunctive singleplex IHCs, are also interpreted in a qualitative fashion by a pathologist using light microscopy. There are many multiplex marker combinations commercially available (see e.g., BioCare Medical, Cell Marque, Mosaic Laboratory websites), and for ease of application to a wide variety of clinical scenarios most currently available kits consist of adjunctive diagnostic markers (United States FDA risk class I).

Assessment of marker coexpression (within cells) and colocalization (by x-y pixel value coordinates) with current chromogenic IHC methods can be challenging. For singleplex DAB/IHC stains, it is usually straightforward to discern whether specific *populations* of cells (e.g., cancer cells, mononuclear inflammatory cells, vascular cells) are positive or negative for each marker. However, due to the requirement for IHC of one section per marker and the size of most cells (∼10 μm) relative to typical section thickness (4–5 μm), it is difficult to discern whether specific *cells* seen in an adjacent H&E-stained section are positive or negative for a given marker, and impossible to tell whether such cells viewed in the original H&E section are positive for more than two IHC markers. For current multiplex IHC assays that precipitate chromogens in tissue, when two different markers colocalize to the same subcellular compartment in the same cells, most commonly with brown and red chromogens, marker colocalization is easily overlooked. This is because mixing light-absorbing chromogens generates dark signals that to the human eye can mimic dark staining of individual chromogens, and overlapping absorption spectra of many chromogens can confound digital image collection and analysis. Another challenge is determination whether lack of marker colocalization is genuine or due to technical interference based on assay technology or design. Moreover, in triplex or higher-plex chromogenic assays, even when markers localize to completely different cell populations, it is difficult for the human brain to comprehend the multicolored patterns and to accurately quantify cell intensities and proportions of positive cells for each marker. Confidence in visual recognition of marker colocalization may be further compromised by microscope setup as well as the limited sensitivity and dynamic range of chromogenic assays. Thus, current singleplex and multiplex chromogenic IHC technologies offer only a limited capability to assess multiple marker colocalization in specific cells. Moreover, as the need to define newly recognized cell phenotypes characterized by simultaneous expression of multiple markers increases, comprehension of stained tissue sections will require automated image acquisition (slide scanning) and software-assisted marker visualization and interpretation.

### Immunotherapy, the Tumor Microenvironment and Multiplex Staining

In the past decade, immunotherapies have transformed oncology research and clinical practice while revealing the importance of endogenous immune “checkpoints” such as PD-L1 and CTLA4 that prevent cytotoxic T lymphocytes in the tumor microenvironment (TME) from targeting a variety of hematological and solid malignancies (Couzin-Frankel, 2013). Despite widespread use of predictive singleplex PD-L1 IHC tests to enrich for likelihood of response to PD-1/PD-L1 axis blockade, the majority of patients do not benefit, and effectiveness is limited in several cancer types likely due to a highly immunosuppressive TME or lack of tumor-specific antigens (Xu-Monette et al., 2017; Hack et al., 2020). By contrast, ipilimumab, targeting CTLA4, does not have an accompanying predictive IHC CDx, although expression of MHC class I has been associated with response to ipilimumab in melanoma (Rodig et al., 2018). Immunoscore® (HalioDx) is an image analysis-based IHC assessment of CD3 and CD8 positive T lymphocytes in defined regions of a tumor biopsy sample, which has shown clinical utility in colon cancer (Angell et al., 2020; Bruni et al., 2020). In recognition of this and other work, tumor immune microenvironment has been added as a prognostic factor by the WHO tumor classification of colon cancer (Digestive System Tumours, 2019). It is important to note that development of Immunoscore preceded successful blockade of immune checkpoint targets in human (Galon et al., 2006), so these and other immune checkpoints as well as other TME immune cell phenotypes of known importance are not assessed by Immunoscore. A better understanding of the interactions among tumor, immune cell subsets, immune checkpoint pathways and other cell types in the TME, including response and resistance mechanisms, will be crucial to develop effective cancer therapies.

The TME is a complex ecosystem consisting of tumor cells, endogenous and tumor-induced stromal cells, vasculature (including vascular endothelia, pericytes, and perivascular cells), nerves and other sensory structures, and various organ/tissue-resident and recruited immune cell types as well as non-cellular components of the extracellular matrix such as collagen, fibronectins, and proteoglycans (Li et al., 2021; Rameshbabu et al., 2021; Vitale et al., 2021). The TME promotes tumor stem cell renewal, proliferation, invasion, and angiogenesis while creating an immunosuppressive environment (Nicholas et al., 2016; Najafi et al., 2019). In solid tumors, dense stromal collagen (desmoplasia) creates a physical barrier that supports cancer growth, in part by promoting hypoxia and precluding entry of immune cells into the tumor mass while maintaining blood vessels that allow tumor cells to metastasize (Mortezaee, 2021), a particularly common feature of pancreatic cancer (Bulle and Lim, 2020). Other components of the TME, such as tumor-associated macrophages (TAMs) (Vitale et al., 2019) and intercellular signals such as IL10 (Ouyang and O’Garra, 2019) and TGF-β (Ganesh and Massagué, 2018) represent therapeutic targets responsible for primary resistance to immune checkpoint blockade (Bulle and Lim, 2020).

Recent advances in cell profiling technologies, data analysis, and visualization tools have unveiled a hitherto unappreciated complexity of the TME and its constituent cell phenotypes (Galon et al., 2006; Digestive System Tumours, 2019; Angell et al., 2020; Bruni et al., 2020). As many TME cell phenotypes most relevant to immuno-oncology are defined by simultaneous detection of more than two markers, singleplex IHC panels will be inadequate to unambiguously identify these cell types in a single tissue section. Several technologies have recently been employed to characterize the TME in research investigations, including multiplexed immunohistochemistry (mIHC) and immunofluorescence (mIF) (Hofman et al., 2019; Parra et al., 2019), mass spectrometry (IMC/CyTOF, MIBI) (Baharlou et al., 2019), single-cell RNA sequencing (scRNAseq) (de Vries et al., 2020), and spatial transcriptomics (Ji et al., 2020).

Several recent studies have employed multiplex methods to investigate the relationship between TME and treatment efficacy as part of exploratory or retrospective analyses of tissue biopsies from clinical trial cohorts. Chaudhary et al. evaluated both short‐ and long‐term effects of prexasertib, a CHEK1 checkpoint kinase inhibitor, on TME of head and neck squamous cell carcinoma, coupling transcriptomics with multiplex mIHC (Chaudhary et al., 2021). Acutely, treated tumors demonstrated increased expression of T‐cell activation and immune cell trafficking transcripts and decreased expression of immunosuppression-related transcripts, but over the longer time points there was an increase in immunosuppression-related transcripts suggesting evasion of immune surveillance that correlated with acquired prexasertib resistance. Schwarze et al. used IHC and mIF on cancer biopsies from a phase IB trial of immune checkpoint inhibition combined with administration of myeloid dendritic cells, revealing treatment-related immune cell infiltration into tumor (Schwarze et al., 2020). Sathe et al. integrated scRNAseq with mIHC to demonstrate dramatic increases in exhausted and regulatory T lymphocytes in gastic carcinoma compared to normal mucosa (Sathe et al., 2020). Gundle et al., in reporting microdosing of drug combinations in soft tissue sarcoma (STS), used mIHC and GeoMx Digital Spatial Profiling (Nanostring) to reveal putative mechanisms of tumor resistance to drug treatment (Gundle et al., 2020). These studies highlight the power of multiplex analysis to reveal a variety of immune cell phenotypes and their spatial arrangements in the TME from a single cancer biopsy, and based on these reports we anticipate growing use of multiplex technologies to probe patient tumor biopsies.

### Multiplex Technologies and the Path From Research to the Clinic

We hypothesize that multiplex technologies most likely to reach clinical application, at least initially, will need to fit in existing histopathology sample workflow with results able to be viewed and interpreted on computer monitors. Many multiplex technologies use fluorescence emission as a means of marker visualization, with some combination of simultaneous and/or cyclic sequential labeling and detection (Lin et al., 2018; Tan et al., 2020). Because fluorescence microscopy is a mature research technique that is already used for a limited number of clinical applications, we believe fluorescence detection will be best suited for initial clinical use.

#### Diagnostic fluorescence microscopy in use today

The use of fluorescence microscopy in routine diagnostic anatomic pathology is currently limited to DNA ISH to detect chromosomal abnormalities (translocations, gene amplifications) and to antibody-based investigations of specific immune and genetic diseases in dermatopathology and nephropathology. A fluorescence microscope and its accompanying viewing monitor are typically located in a darkroom, outside of the main lab, in order for users’ eyes to accommodate viewing images with a dark background. In contrast, the background of the H&E or DAB-stained image viewed in a brightfield microscope is usually white, and such images can be comfortably viewed for hours in a brightly lit room. Typically, dedicated technicians gather images from fluorescent diagnostic assays (e.g., DNA FISH) for the pathologist to review for case sign-out, freeing the pathologist from the dark room. One solution to the “dark room” problem is for the pathologist to review and interpret fluorescent WSI, possibly with false coloring or color inversion to create an artificial white background on a computer screen. Such images can be obtained from whole slide fluorescence scanners, that, unlike the fluorescent microscope, are not required to sit in a darkroom, but rather feature automated workflows and high throughput for enhanced viewing and analysis on computer monitors.

#### Differences between brightfield and fluorescence microscopy

In addition to these practical differences between fluorescence microscopy and brightfield microscopy, there are important differences in the relevant laws of physics that underlie viewing stained tissue by each type of microscopy. To the detector, whether a camera or the human eye, brightfield microscopy measures an absorption process (subtraction of light), while fluorescence microscopy measures an emissions process (addition of light). Radiative transfer, which accounts for the emission and transport of electromagnetic radiation (light) through a medium, confers significant advantages for fluorescence over brightfield imaging in terms of dynamic range, sensitivity, and the ability to measure multiple signals at once (multiplexing):

#### Dynamic range

With chromogenic staining and brightfield microscopy, transmitted light passes through the sample, and light intensity in each “column” of absorption [i.e., tissue thickness (z) at each x-y coordinate of the tissue plane] is inversely proportional to the abundance of deposited chromogen. Once the absorption column has become optically thick (e^−τ^ where the optical depth, τ > 1), the ability to detect additional chromogen in a heavily stained column becomes exponentially more difficult (i.e., the stain “shadows” itself). Consequently, with heavy staining, section thickness and enzyme reaction time can dramatically impact perceived stain intensity and can create challenges in consistently distinguishing between moderate vs. strong staining in semiquantitative IHC assays. In addition, chromogen diffusion can leave a lightly stained “diffusion halo” of several hundred nanometers or more around intensely stained structures (more prominent with fast-red based detection by alkaline phosphatase). In areas with low levels of chromogenic staining, focal plane, objective lens magnification and numerical aperture, and other features of the optical system can impact detection sensitivity. A recent study on focus standardization of H&E-stained WSI, obtained from different scanners, revealed that a substantial amount of out of focus information is retained by an “in focus” brightfield image (Kohlberger et al., 2019). Fluorescence imaging uses a completely different method to detect marker abundance, with the intensity of the emission column at each x-y position in the sample being directly proportional to amount of the fluorophore in the column. Because a thin tissue section is nearly optically transparent, essentially all emitted light from fluorophores passes easily through the column and is detected by the camera. As discussed above, colocalization of chromogenic dyes increases the darkness of tissue when viewed by brightfield microscopy, whereas with fluorescence based detection overlapping signals become brighter with increased marker abundance. These differences in the physics of brightfield vs. fluorescence imaging contribute to the fact that, in practice, HRP/DAB is limited to ∼2 orders of magnitude of dynamic range while fluorescence can detect ∼5–6 orders of magnitude, approaching the intrinsic dynamic range of the protein concentration in biological specimens of ∼7 orders of magnitude or more (Rimm, 2006; Zimak et al., 2012; Vani et al., 2017).

#### Sensitivity

In fluorescence imaging, to achieve higher sensitivity one can increase the excitation light intensity to further increase the flux of emitted light and/or increase camera exposure time to collect additional signal. Autofluorescence of some FFPE tissues can limit sensitivity by increasing background emissions at different wavelengths (Lazarus et al., 2019). With chromogenic imaging, above a certain level of absorption there is little sensitivity gained with brighter illumination. The fluorescent signal can also be amplified by introducing more fluorophores per antibody in the staining assay (Zimak et al., 2012), while for chromogenic staining, adding more absorbing molecules has a fast-diminishing effect once the optical depth of the stain column is above a certain amount.

#### Higher-order marker multiplexing

Higher order multiplexing is possible for fluorescence imaging because the absorption and emission spectra of fluorescent probes are generally narrower than those of chromogenic stains. Given the finite bandwidth of the optical spectrum, this property allows for a greater number of multiplexed signals to be simultaneously detected—typically five fluorescent channels with conventional filter sets and up eight or nine channels with special filter sets and “spectral unmixing” (defined below) as compared with two or three simultaneous colocalized colors in a chromogenic image. DAB, the most commonly used chromogen, has a broad transmission spectrum overlapping with red and yellow (Gordon, 1988), making it difficult to accurately quantify DAB when other chromogens are present. Spectral unmixing is a mathematical operation, a nonlinear least-squares fit, that estimates the proportion of each fluorophore’s contribution (and any tissue autofluorescence) to the overall spectrum at each wavelength when there is spectral overlap (spectral bleed-through) (Dickinson et al., 2001). But, when considering potential diagnostic uses of fluorescence, a requirement for spectral unmixing in the detection system may compromise consistent tracing of information through the so-called “pixel pathway” - the framework, described below, that governs how regulatory bodies view WSI systems for diagnostic use (Abels and Pantanowitz, 2017).

##### Standardization of multiplex immunofluorescence workflows.

Fluorescence microscopy as a technique is far less standardized than brightfield microscopy, with each microscope manufacturer offering distinct lens materials, light sources, optical paths, filter and mirror sets, detection cameras, and viewing software. With mIF, microscopists can generate images that maximize signal to noise ratios for the given marker, antibody clone, fluorophore, tissue/sample type, preparation method, strength of emission light, camera exposure time, and experimental aim—all too frequently with only the goal of generating a beautiful and visually striking image for a publication, journal cover, or marketing material. However, these parameters require simultaneous optimization to achieve an optimal result—which might be very different for the next experimental condition, set of tissue samples, equipment setup, or laboratory. There are increasing options for whole slide fluorescence scanners that create multiplex WSIs, but these are not yet standardized with respect to how images are generated or how the output files are formatted. A critical aspect of diagnostic development and validation, even in a single laboratory, is defining the diagnostic system, locking it down, and then testing performance on scaled sample sets in relation to the assay’s expected use. Thus, one major challenge of implementing fluorescence-based tissue marker detection in clinical practice is defining the best system parameters from a wide variety of system components and configurations so that the fluorescent images can be compared to ground truth, typically “gold standard” brightfield IHC images.

Thus, for diagnostic use mIF has numerous potential advantages over enzyme-based chromogenic staining, allowing simultaneous detection of multiple markers in individual cells and reducing the number of tissue sections necessary for complete assessment of markers currently tested with IHC. As noted, this may be advantageous in situations where diagnostic tissue is limiting, such as lung cancer. Most importantly, as deep profiling methods (transcriptomics, proteomics) are used to probe individual cells in normal and diseased tissues, most notably as part of the Human Cell Atlas (Regev et al., 2017), it should be possible to specify a standard, minimal set of markers to unambiguously identify specific and well-defined pathogenic cell types and their locations using mIF on tissue biopsies.

*Phases of multiplex immunofluorescence testing.* A useful framework to consider mIF assays is the preanatlytic, analytic, and postanalytic phases of testing. For DAB/IHC assays, the analytic phase is staining itself, either manually by a technician or by an autostainer. Preanalytic factors include all steps from sample procurement to staining, including fixation, processing, embedding, slide preparation, and any manual tissue pretreatment. It is estimated there are over 100 discrete steps in the preanalytic phase, and beyond formalin fixation past a certain time (e.g., 8 h for ER/PR IHC of breast biopsies per CAP recommendations), practices are not standardized and thus vary widely (Agrawal et al., 2018; Compton et al., 2019). Importantly, antigen retrieval steps that lyse formalin cross links and expose epitopes prior to staining allow many singleplex IHC assays to retain robust assay performance, at least for qualitative interpretation, despite variation in preanalytics (Bogen et al., 2009). The analytic phase on the autostainer includes any automated pretreatments, antibody blocking and incubation steps, washes, and enzyme-based signal detection. The postanalytic phase includes applying the slide coverslip, any post-run slide labeling, and interpretation by the pathologist. Variation in any of these test phases can cause variation in results, as well as false positive or false negative results. Over 4 decades of practice experience with DAB-based IHCs has led to improved diagnostic assay standardization that—considering disparate reagent sources and automation platforms – allows for some degree of comparability between assays across antibody clones, platforms, and laboratories. Such inter-assay comparability is emphasized by FDA in IHC guidance documents and IHC-based product approvals (Guidance for Industry, 1998) as well as CAP-recommended updates in assay interpretation [e.g., for the Her2 IHC and FISH assays (Wolff et al., 2018)]. mIF workflows are far less standardized, and quantitation of images requires additional analytic and post-analytic steps such as fluorescent slide scanning and image capture, image processing and analysis, and viewing on a computer monitor. Thus, for mIF the diagnostic workflow is expanded relative to traditional IHC, such that the postanalytic steps of staining become the preanalytic steps for slide scanning and analysis. Tissue and slide quality impacts scan quality, which can vary widely between vendors, models, and laboratories; scan quality in turn influences image analysis (Dunstan et al., 2011; Webster and Dunstan, 2014) as well as performance of AI algorithms (Cui and Zhang, 2021).

*Multiplex fluorescence technologies.* Several multiplex assay platforms, technologies, and protocols have been recently reviewed (Lin et al., 2018; Hofman et al., 2019; Francisco-Cruz et al., 2020; Tan et al., 2020; McGinnis et al., 2021). Traditional mIF assays use fluorophores directly conjugated to primary or secondary antibodies. With traditional IHC, sensitivity is enhanced by increasing the number of HRP molecules per primary antibody, such as by avidin/biotin complexes or HRP-polymers. The same principle holds for mIF, with sensitivity enhanced by increasing the number of fluorophores per primary antibody molecule, allowing generation of quantitative data across analyte concentration ranges that reflect relevant physiologic or pathological states in tissue (Zimak et al., 2012). In newer mIF methods, application and detection of antibodies can be sequential, simultaneous, or some combination thereof. Some methods can be performed manually, but recent data suggests automation improves precision and reproducibility (Surace et al., 2019; Taube et al., 2020), performance attributes that will be essential to build confidence in diagnostic use. In each method, specific fluorophores need to be matched to antibody/target molecule, emissions spectra, filter sets, camera settings, tissue type, and proposed data analysis pipeline. A variety of mIF methods, including hapten-based, cyclic tyramide-based amplification and DNA barcode-based detection allow higher sensitivity and higher order multiplexing beyond the traditional species barriers imposed by secondary antibody-based detection. Emerging methods such as CODEX (Akoya, Inc.) (Goltsev et al., 2018), MACsima (Miltenyi Biotec), Orion (Rarecyte, Inc.), GeoMx Digital Spatial Profiling (Nanostring, Inc.) (Toki et al., 2019), and Visium FFPE (10x Genomics) generate higher-plex spatial analysis (20–40 or more markers on a single section), but we speculate these techniques are better suited for discovery rather than immediate clinical applications until challenges associated with long turnaround times, high cost per sample, sample destruction, and stringent validation requirements are overcome.

Among the most widely used mIF method, especially in immuno-oncology, is Tyramide Signal Amplification (TSA) (Opal, Akoya, Inc.), a cyclic staining protocol using tyramide-conjugated fluorophores (Stack et al., 2014). Briefly, the TSA method amplifies fluorescent signals through a polymer-HRP detection system similar to traditional IHC, but instead of using DAB to deposit chromogen, HRP activates tyramide to covalently bind multiple tyrosine residues near the epitope of interest. Non-covalently bound antibodies are then stripped using heat, while tyramide-linked fluorophores accumulate on the tissue with each cycle of staining. This staining/amplification cycle is then repeated up to 7 more times (generating up to an 8-plex image) with different antibody/fluorophore combinations, with consideration to order of target detection as well as rigorous controls required during assay development and with each experiment to ensure accurate detection of each marker. Once an assay is developed, the advantages of this technique are its simplicity, enhanced sensitivity relative to fluorophore-linked secondary antibodies, high specificity, and compatibility with most fluorescent microscopy systems. Spectral unmixing, discussed above, is an obligate requirement in the Opal workflow. With multiple rounds of epitope retrieval, tissue integrity may become compromised, limiting assay plexy and precluding use of the mIF slide for additional assays such as H&E staining. A recent improvement in the TSA method using a stripping buffer instead of heat underscores the importance of maintaining tissue integrity for any mIF analysis (Willemsen et al., 2021).

**FIGURE 1:**
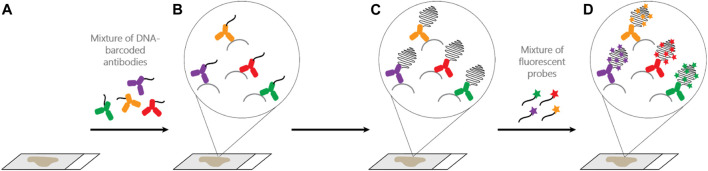
Ultivue InSituPlex (ISP) assay workflow. **(A**,**B)**: Four different DNA-barcoded antibodies are applied to a FFPE tissue section. **(B**,**C)**: Barcode amplification. **(D)**: Targets are labeled through hybridization of fluorescent probes to their respective barcodes. Higher plex staining (greater than 4-plex) is possible by initial application of all antibodies, each with a unique barcode, to the tissue and then detecting 4 markers per cycle, separated by gentle DNA exchange steps (not shown).

**FIGURE 2:**
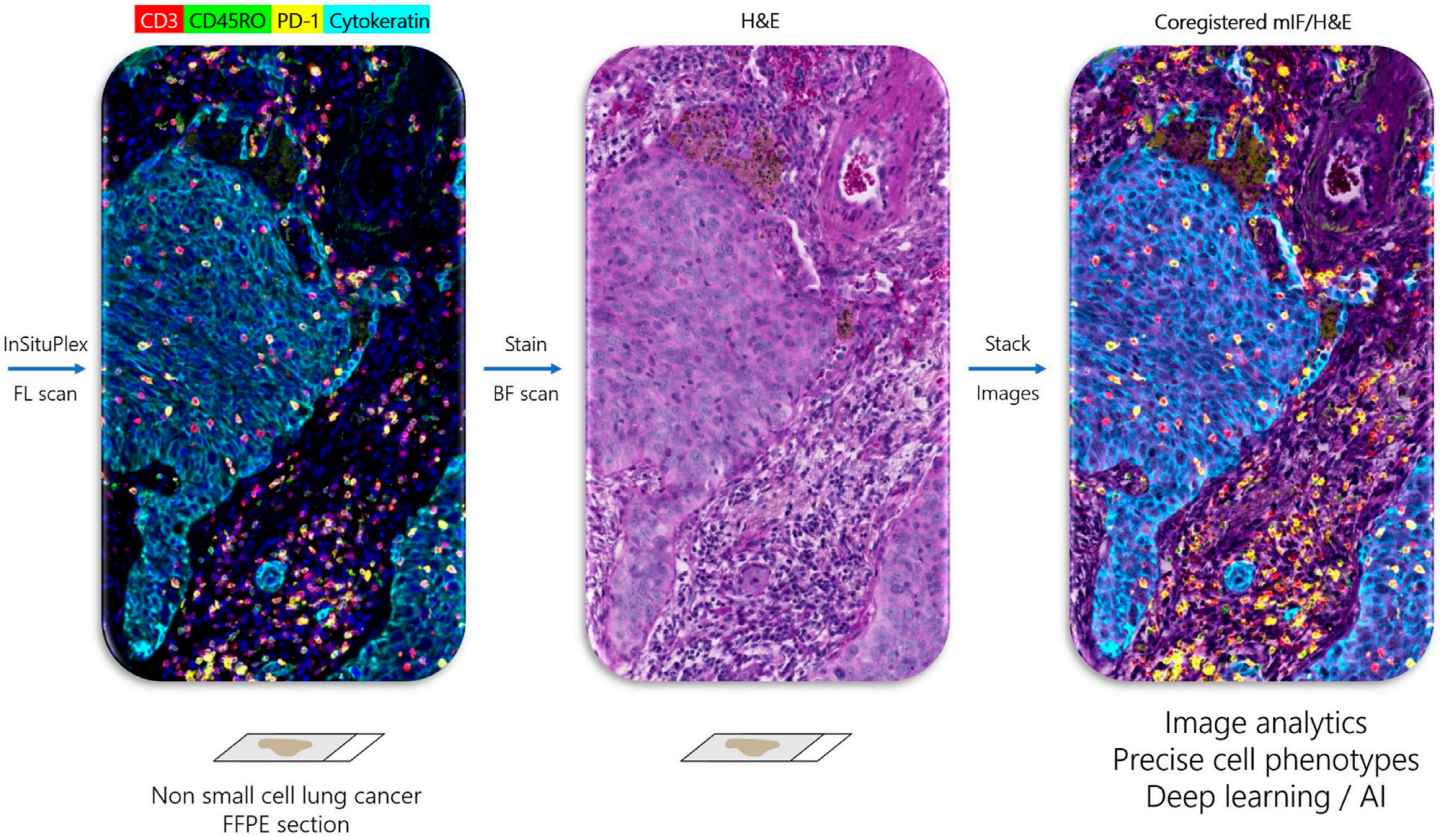
Ultivue InSituPlex workflow enables coregistration of multiplex IF and H&E tissue images. Left panel shows image of non-small cell lung cancer stained with four-marker panel including CD3^+^ T cells (red), CD45RO+ memory T cells (green), PD-1+ exhausted T cells (yellow), and Cytokeratin positive carcinoma cells (cyan) obtained by fluorescent (FL) scan. H&E stain on same section (middle panel) obtained by brightfield (BF) scan. Right panel is coregistered overlay of multiplex and H&E images, which allows assignment of marker phenotype to every cell in the H&E section, which may be used for integrated image analytics and training of deep learning networks.

One recently developed method with several advantages over cyclic TSA-amplification is Ultivue’s InSituPlex (ISP) technology (Figure 1). Antibodies against four different targets are each conjugated to a unique DNA barcode sequence. After a single antigen retrieval step, all antibody-DNA conjugates are applied to the slide. The barcodes on each antibody are then amplified *in situ*, avoiding secondary antibodies that can exhibit unwanted cross-reactivity. Next, fluorescent probes complementary to each barcode label each target, enhancing sensitivity. ISP has been automated on autostainers (BOND RX, Leica Biosystems), and slides can be imaged on a variety of fluorescent scanners and analyzed using any image analysis software. ISP can be performed in multiples of 4-plex (e.g., 8, 12, 16 plex) by applying all barcoded antibodies simultaneously and then detecting four fluorophores (plus nuclear counterstain to detect DNA and mark cells) per cycle. ISP features a rapid, low-complexity, easily automated workflow with pre-optimized, highly sensitive assays that can deliver reproducible results comparable to other methods (Humphries et al., 2020), but with high throughput and faster assay development times than TSA-based assays. Because ISP uses standard, gentle antigen retrieval, following mIF staining the slide can then be stained for H&E and the resulting WSI precisely merged with mIF data, allowing association of every cell in the H&E section with its marker profile (Figure 2).

*Quantitative analysis of multiplex fluorescence images.* Image analysis software is required to analyze the massive datasets created by whole slide mIF. A subdiscipline of computer vision, image analysis can be applied to WSI to quantify marker pixel counts or structures in regions of interest (such as tumor vs. nontumor) selected manually or in an automated fashion using marker status or AI. Various cell types, defined by the presence or absence of one or more markers, can be quantified in the tissue section by number, location, density, proximity to other cell types or structures, or any other metric of interest. Tissue image analysis using rules-based machine learning algorithms has been used for decades in research, yet the few FDA-cleared clinical applications for image analysis of IHC are mostly restricted to algorithms that quantify CDx markers such as Her2 and ER/PR (Abels et al., 2019; Aeffner et al., 2019; Zuraw et al., 2020; Digital Pathology Association, 2021). Recently, deep learning-based AI algorithms have been employed to analyze large collections of H&E tissue slides to identify tissue features such as various types of cancer and to predict molecular lesions such as specific gene mutations (Couture, 2019; Chen et al., 2020; Rana et al., 2020; Echle et al., 2021; van der Laak et al., 2021), providing proofs of concept that image analysis can detect information in WSIs that are not detectable by a trained human observer. We hypothesize that unambiguous assignment of multi-marker cell phenotype to every cell in the tissue section, achieved by precise coregistration of H&E and mIF images, will augment interpretation of the H&E section, whether read by a human pathologist or an AI algorithm.

*Regions of interest.* One major area of relevance to cancer diagnostics concerns specification of regions of interest (ROI) in each tissue sample. Pathologists use (and are legally required to view) representative sections of all stained tissue blocks, typically by H&E and IHCs, to render a diagnosis; to do otherwise, by intentional or unintentional omission of tissues for review, increases likelihood of misdiagnosis and constitutes grounds for malpractice. For TME assessment in immuno-oncology, how many and which areas of the slide to analyze remains largely undefined. Beyond the categories of hot, warm, and cold tumors based on location and density of inflammatory infiltrates (Bonaventura et al., 2019) - more recently referred to as inflamed, immune excluded, and immune desert, respectively (Hegde and Chen, 2020) - many tumors exhibit heterogeneity and intermediate attributes between these categories, and even tumors classified as “hot” exhibit heterogeneity unrecognized by traditional IHC analysis (Shembrey et al., 2019). To accommodate such heterogeneity and estimate critical parameters, pathologists have historically relied on identification of relevant random or defined “fields of view” (FOVs), such as when estimating mitotic rates to grade sarcomas (Neuville et al., 2014). Should TME analysis be based on the whole slide or on specific FOVs? If the latter, how many FOVs, and how should they be selected: randomly, with consideration paid to tumor architecture, or by specific criteria? For example, since the invasive front is a region where tumor cells can be visualized interacting with adjacent non-neoplastic tissue, it seems intuitive that invasive front FOVs should be analyzed to estimate risk of metastasis (Eiro et al., 2012). However, different driver mechanisms and intercellular interactions may be operative within the tumor mass and its invasive front, and different mechanisms may be dominant in different areas of the invasive front (Lawson et al., 2018), so either the entire invasive front should be analyzed, or FOV selection should be guided by criteria linked to the pathogenic mechanism being evaluated or by less biased image analysis or AI techniques. In either case, we believe WSIs will need to serve as input for FOV selection, whether chosen by a human pathologist or by computer software.

**FIGURE 3:**
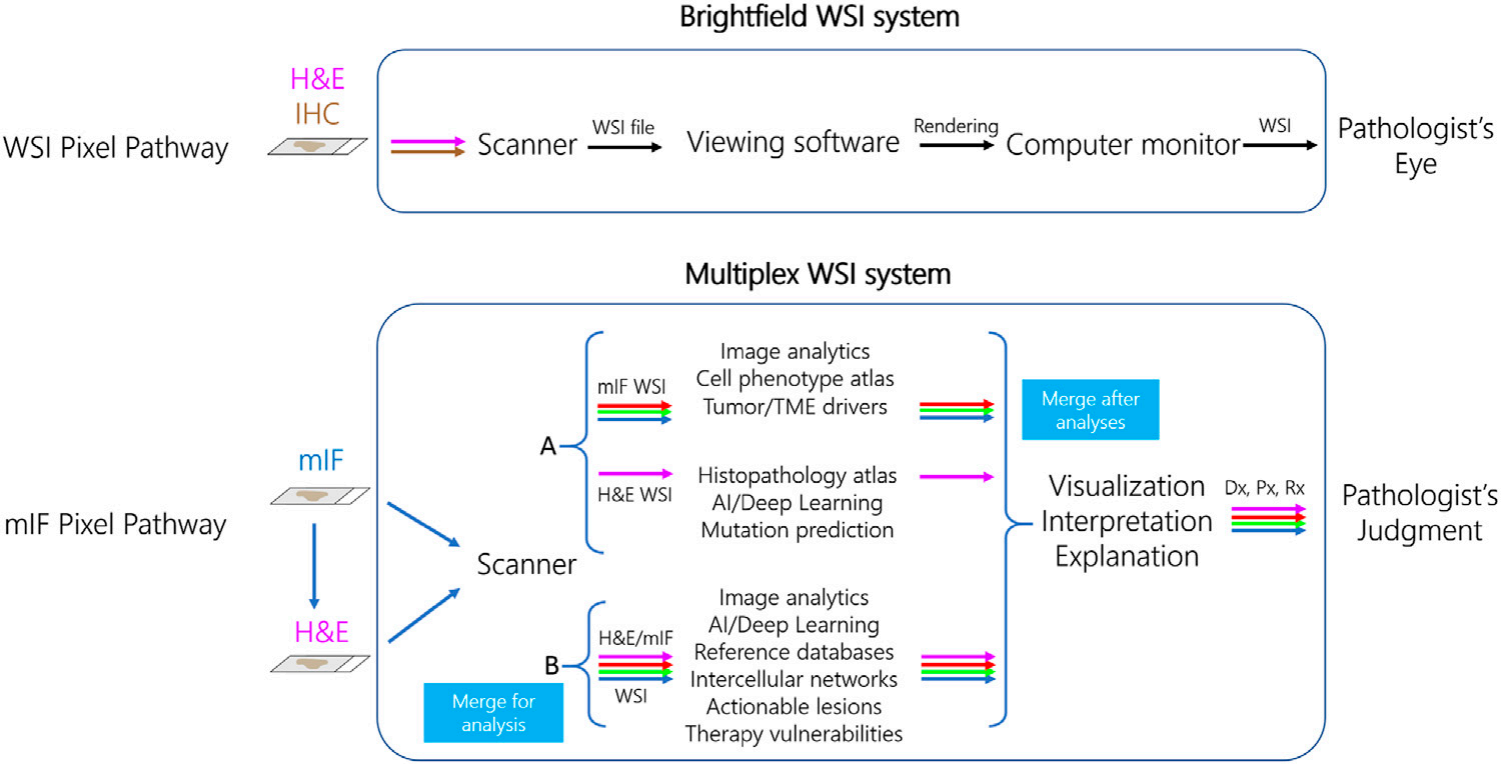
Pixel pathways for diagnostic whole slide imaging (WSI) and multiplex immunofluorescence (mIF) systems. In the current FDA WSI system paradigm **(top row)**, image pixels are traced sequentially through a three-component system that includes brightfield scanner, viewing software, and computer monitor to be interpreted by the Pathologist. In a proposed cell-phenotype centric mIF system **(bottom row)**, the pixel pathway originates with scanning of mIF and then H&E stains on the same slide, a capability of Ultivue’s ISP technology. In model A **(top)**, mIF and H&E images are separately analyzed using the indicated tools, and then images are merged after analysis. In model B **(bottom)**, merged mIF and H&E images are subject to a novel data pipeline such as cell-based annotation of H&E sections for analysis by deep learning, allowing earlier definition of each cell’s phenotype in the system’s pixel pathway. Both models converge so the Pathologist can visualize the merged image with analyses, followed by judgment of the diagnostic (Dx), prognostic (Px), and predictive (Rx) explanations and interpretations when issuing the patient’s diagnostic report.

*Whole slide imaging systems for diagnostic use and the “pixel pathway.”* When considering how mIF might enter diagnostic practice it is important to consider how health authorities such as the FDA have approached regulation of WSI systems using brightfield microscopy. Over 2 decades ago, whole slide scanners that create high resolution images from standard pathology tissue slides were marketed for research and educational purposes, with the perceived promise (and among some, fear) that they would someday augment or even replace manual microscopy in diagnostic practice. Such was born, as an extension of efforts around telepathology for remote diagnosis in the 1980s and 1990s, the field of “digital pathology” (Soenksen, 2009; Weinstein et al., 2019). Slide digitization enabled quantitation of tissue parameters by image analysis, described above. However, at that time there were no digital pathology systems approved as IVDs for primary diagnosis by FDA. Several years of negotiations between the Digital Pathology Association (DPA), College of American Pathologists (CAP), various scanner manufacturers, and the FDA eventually led to WSI system definition and performance standards that could be used as a basis of FDA approval (Abels and Pantanowitz, 2017). For a WSI system to achieve FDA clearance for diagnostic use, it was agreed that over 2000 cases of the variety seen in a typical surgical pathology clinical practice needed to be assessed by over a dozen pathologists using many, physically separate (but identically specified) WSI systems. A WSI system consists of three separate but connected devices including the slide scanner, viewing software, and computer monitor (Figure 3) (Abels and Pantanowitz, 2017). At the heart of defining a WSI system for manufacture, distribution, and promotion was its “pixel pathway,” the path of each image pixel as it transited through the WSI system—from the tissue slide to the pathologist’s eye. The pixel pathway concept served several purposes: 1) as an initial step toward WSI system standardization, 2) generation of diagnostic accuracy data to show that diagnoses made using different WSI systems were noninferior to diagnoses made by manual microscopy, and 3) identification of various system failure modes and the potential to attribute system failures (e.g., a false positive or false negative diagnosis) to a root cause such as a specific component of the WSI system itself, the pathologist, or to an intrinsically challenging differential diagnosis such as dysplasia vs. carcinoma *in situ*. To date, only two such WSI systems have been cleared for primary diagnosis by the FDA, the first by Philips in 2017 and the second by Leica Biosystems in 2019 (Mukhopadhyay et al., 2018; Bauer et al., 2020; Borowsky et al., 2020). These and other studies have engendered confidence among pathologists that using WSI is as safe as using their microscope for primary diagnosis. There remain widespread barriers to uptake of digital pathology systems, which will need to be addressed before mIF is accepted as a diagnostic tool. Primary among these is a lack of standardization and interoperability between different WSI components and systems (Marble et al., 2020), meaning that each WSI system has a distinct pixel pathway design. Moreover, for WSI systems to fulfill current standards requires device manufacturers to invest multiple years and millions of dollars in system development, specification, and validation - a long-time frame and large investment compared to the rapid technological advancements and decreasing costs of digital imaging technology and deployment options in individual labs under LDT enforcement discretion. Additional factors that need to be addressed include lack of incentives for digital pathology infrastructure investments and reimbursements (Lasiter et al., 2020), mouse-driven and ergonomically unfriendly “point and click” viewing software (Molin et al., 2015), creation of viable business cases for implementation (Lujan et al., 2021a), and a pathologist’s fear of being tethered to a potentially unreliable computer system as opposed to a trusted manual microscope. Recognition of these issues was accelerated in 2020 by the COVID-19 pandemic, which prompted some institutions to rapidly validate their digital pathology systems for diagnostic use in order to maintain continuity of care (Hanna et al., 2020; Stathonikos et al., 2020; Samuelson et al., 2021; Lujan et al., 2021b), whereas other institutions relied on less rigorous system validation guided by the pathologist’s ability to judge when images are of insufficient quality to make a diagnosis. A notable parallel concerns the application of telecytology (remote microscopic viewing of cytology specimens) for rapid onsite evaluation (ROSE) of adequacy of biopsy specimens, which is recommended to follow CAP guidelines for validation of diagnostic WSI systems (Pantanowitz et al., 2013; Lin et al., 2019; Evans et al., 2021).

*Standardize cell phenotypes before pixel pathways.* It has been proposed that widespread adoption of digital pathology in health care will require ecosystem-wide implementation of standards akin to those that enabled the field of diagnostic radiology to convert from film-based to digital platforms over a decade ago (Herrmann et al., 2018; Clunie, 2021). A pivotal element of radiology’s digital conversion was establishing data format standards and component interoperability standards that serve as a basis of device regulatory approval, such that unique system configurations and workflows can be established at each facility from interchangeable components that will perform in a predictable fashion when combined in a system. While some digital pathology standards exist, particularly around the emerging use of DICOM file formats, regulators and manufacturers have not yet agreed upon standards that can serve as basis of product development, testing, approvals, and marketing (Herrmann et al., 2018). As a result, many digital pathology systems used for primary diagnosis throughout the United States are distinct, classified as LDTs, because each system consists of a unique mix of components that may or may not be approved by the FDA for specific uses.

Given the myriad potential mIF system configurations and potential diagnostic uses, coupled with lack of standards in mIF and in digital pathology in general, we do not foresee all relevant stakeholders agreeing to a standardized “pixel pathway” for diagnostic mIF-based WSI systems any time soon. Instead of focusing on image pixels, since mIF is most commonly used to identify cell phenotypes defined by coexpression of multiple markers, one potential step towards standardization of mIF data would be to first standardize data formats at the level of identification and characterization of each individual cell in the sample, perhaps in a manner analogous to how flow cytometry manufacturers created the Flow Cytometry Standard (FCS) data format (Spidlen et al., 2021), with addition of a cell position coordinate in the x-y tissue plane (including the relevant image patch) specifying the location of each cell in the tissue. Such “tissue cytometry” is not a new concept (Ecker and Steiner, 2004; Blenman and Bosenberg, 2019), but advances in multiplex technology, understanding of single cell biology and the role of pathogenic cell types in disease, increased computational power and AI, and a requirement to better characterize the TME are creating urgency around diagnostic use of multiplex staining. Irrespective of efforts to standardize mIF and digital pathology, each diagnostic mIF system used as a LDT will have a unique “pixel pathway” and data pipeline to identify cell phenotypes that could serve as a basis of in-laboratory verification and validation testing, as well as to identify failure modes and their root causes. Similarly, by analogy to brightfield WSI systems, stringent validation of single-site mIF systems used as LDTs should be far easier than the multisite/multisystem validation required of candidate mIF IVD systems. Nearly all FDA-approved CDx IVD tests, including those based on IHC or ISH, are class III risk class devices requiring premarket approval (PMA); however, an increasing number of FDA-approved CDx assays are classified as LDTs, but only one assay (PDGFRB FISH for imatinib eligibility) is tissue-based, the remainder being PCR or NGS-based (Jørgensen, 2021).

*Use of immunofluorescence data in diagnostics.* How might mIF data be analyzed in future diagnostics? At least two cell phenotype-centric models for mIF WSI system “pixel pathways” can be proposed (Figure 3). In one model (“A” in Figure 3), the H&E section and mIF data are subject to separate analysis pipelines and then merged after analysis. In line with many recent applications of AI to pathology, H&E image pixels and features are linked by supervised training of convolutional neural networks (CNNs) to data such as pathologist-annotated image features, specialist-rendered diagnosis, presence or absence of molecular lesions, prognosis, or treatment outcomes. In parallel, mIF is used to identify and quantify specific cell phenotypes and their locations in the biopsy (Wilson et al., 2021). This model is analogous to the current addition of non-histology-based biomarkers such as NGS panels to pathology diagnosis, with results integrated at interpretation and reporting stages. An alternative model (‘B” in Figure 3) exploits ISP’s ability to generate a merged H&E and mIF image (Figure 2) as input data to generate cell-level annotations of H&E slide images for training CNNs. We envision at least two advantages of model B. First, it eliminates the need for manual pathologist annotations of H&E images for algorithm training, widely viewed as a key limiting factor in global deployment of AI in pathology (van der Laak et al., 2021). Second, identifying the multi-marker profile of every cell in the H&E-stained tissue biopsy results in earlier identification of cell phenotypes in relation to their interpretation appearances and tissue distributions in the system’s pixel pathway. There are a few examples using singleplex IHC to augment annotation of H&E sections for training AI [reviewed by van der Laak et al. (2021)], including use of cytokeratin IHC to aid identification of breast (Litjens et al., 2018) or prostate (Bulten et al., 2019) cancer cells, detection of mitoses using phosphohistone H3 (Tellez et al., 2018), and detection of breast cancer cells using cytokeratin and Ki67 IHC (Valkonen et al., 2020), and one recent example using mIF of tumor infiltrating lymphocyte (TIL) markers to predict driver mutations in colon cancer (Bian et al., 2021). In either model, mIF and H&E data could be merged by the scanner or by analysis software after scanning to be rendered for viewing and interpretation.

As mentioned, in addition to using H&E-stained tissues to train AI to assist with histopathology diagnoses, recent reports have shown that AI can predict a tumor’s mutational status normally revealed by molecular diagnostic tests such as PCR or NGS (Burlutskiy et al., 2020). This has in turned raised the question – heretical to molecular pathologists - of whether molecular tests are necessary for diagnosis. We speculate that given the substantial cost of molecular testing, initial diagnostic uses of H&E interpretation by AI may serve to increase the diagnostic yield of molecular testing by screening out cases likely to yield a negative result (high sensitivity with 100% specificity). Similarly, it is unknown whether mIF will ultimately be necessary for diagnosis, i.e., whether specific cell phenotypes such as pathogenic macrophage subtypes or regulatory T cell subtypes currently identified only by multiplex methods can be recognized in H&E sections alone by properly trained AI, or whether multiplex staining will be required to identify such cells in every biopsy. Since H&E-based interpretation is so critical to diagnostic pathology, we anticipate that irrespective of the pixel pathway model of the mIF system, the pathologist will require direct coregistration of the H&E with the multiplex images so they can visualize complex cell phenotypes on the same tissue section they use to make the primary diagnosis.

*Preparing for multiplex in clinical practice.* Given the established use of IHC to detect markers in routine anatomic pathology, the promise of multiplex tissue analysis as a basis of new diagnostics, and the current regulatory landscape of digital pathology, we offer the following conjectures:

1) Unlike singleplex IHC, the complexity and diagnostic significance of multiplex data used to identify multi-marker cell phenotypes cannot be grasped by the pathologist without computational assistance. Therefore, pathologists must be trained on digital pathology software to visualize, quantify, and interpret multiplex tissue data.
2) Multiplex data needs to seamlessly integrate into the digital pathology work environment used for primary diagnosis, including integration with H&E-stained slide images, molecular studies, and other patient and slide metadata.
3) Manual fluorescence microscopy is not a preferred diagnostic modality for pathologists, meaning that expanded use of fluorescence detection in clinical practice will require a scanner to generate WSIs that can be viewed, manipulated, and analyzed by digital pathology software.
4) Interpretation of mIF data will require image analysis, augmented by explainable AI algorithms, to understand and interpret data and report diagnoses (Huss and Coupland, 2020; van der Laak et al., 2021).
5) Until Pathology adopts data formatting and component interoperability standards akin to Radiology, including integration of multiplex tissue data, end-to-end WSI systems used in a diagnostic capacity will likely be custom applications operating as LDTs in each laboratory site.
6) By analogy to brightfield WSI systems, health authorities such as FDA will require mIF WSI IVD systems to have a defined pixel pathway, from slide staining to stain visualization and interpretation. Fundamental differences in brightfield vs. fluorescent microscopy, the lack of standards around mIF systems, protocols, data collection and software analysis pipelines (especially if the WSI system provides decision support as a medical device), and a requirement for similar clinical interpretation across platforms and practice environments, implies that establishing standards to achieve clearances for a mIF WSI system (or even a for a stand-alone scanner) will be challenging.
7) Since a major goal of mIF analysis is to assess multiple marker colocalization in cells to identify and score specific cell phenotypes, we propose that efforts to standardize data should first focus, by analogy to flow cytometry, on standardizing definitions of specific cell phenotypes rather than on striving to create a standardized mIF pixel pathway.
8) Precise multi-marker annotation of every cell in the H&E slide by mIF data will augment training and performance of AI on H&E-stained tissue samples for some but not all relevant elements of tissue diagnosis. Optimal uses of AI on mIF data in diagnostic workflows have yet to be defined (Mungenast et al., 2021).
9) Pathologists must have greater access to digital pathology systems and software including image analysis/computational pathology tools in order to begin to integrate multiplex analysis of any kind into primary diagnosis.

### Imagining the Pathologist’s Future

As WSI scanners and viewing software gained a dedicated user base, the concept of the “pathologist cockpit” emerged as a model for digital pathology-based case sign out of the future (Soenksen, 2009). Just like an airplane cockpit, it was imagined that all the controls, dials and knobs, sticks and gadgets necessary for the pathologist to navigate from point A (tissue intake) to point B (the diagnostic report) would be laid out on multiple screens. A decade later, many centers have created multiscreen pathologist cockpits that bring pathology data and relevant software to the pathologist’s fingertips. We can now imagine, in outline and with some detail, the pathologist’s cockpit of the future: multiplex profiling will identify the phenotype of every cell in H&E tissue section; vast computational power will enable access to knowledge databases such as cell and tumor atlases; and AI will help the pathologist make sense of it all to better help clinicians select the best therapy for their patients. We expect that the next generation of pathologists, like sages on mountaintops, will be ever the wiser with an expanded ability to navigate disease.

## Conclusion

Rules and regulations governing creation and deployment of diagnostic tests are of necessity geared to ensure patient safety and preserve equipoise in clinical investigations (Rabinstein et al., 2016; O'Neill et al., 2019), but regulations can also oppose innovation, thereby denying patient benefit. The potential of digital pathology to transform anatomic pathology practice is not limited to remote case sign-out or training AI to interpret H&E slides; its potential will be realized when knowledge about single cell phenotypes and disease driver pathways, unique to each patient and their disease and revealed by multiplex marker labeling methods, is available for every pathologist to interpret every patient’s tissue biopsy. IHC has been a major contributor to understanding the roles of single cells and cell populations in diagnostic biopsies, but as currently practiced only allows interrogation of one marker, one molecular species at a time, and is incapable of identifying emerging cell types of importance defined by coexpression of multiple markers in the same subcellular compartment. Given the current regulatory landscape of diagnostic anatomic and digital pathology, the technical demands of multiplex assays, and lack of standardized mIF methods, we propose that retrospective analysis of clinical trial cohorts and development of diagnostic assays as LDTs in individual laboratories will increase assay confidence and generate real world evidence of clinical validity and, by inference clinical utility, which in turn will inform the optimal design, performance and testing of standardized diagnostic multiplex systems of the future.
